# Female sex workers and condom use: lessons for future HIV vaccine trial in Nigeria

**DOI:** 10.1186/1742-4690-9-S2-P224

**Published:** 2012-09-13

**Authors:** O Fakunle, A Adeyemi, O Busari, O Adesola

**Affiliations:** 1 University College Hospital, Ibadan, Nigeria; 2 MEASURE, Abuja, Nigeria; 3 Federal Medical Center, Ido-Ekiti, Nigeria; 4 Mainland Hospital, Lagos, Nigeria

## Background

Condom remains one of the most effective means of HIV prevention in the absence of proven, safe and effective vaccine. Nigeria has a surveillance system that monitors HIV trend among most-at-risk populations. Female sex workers (FSW) have the highest HIV prevalence compared to other groups. Since the ultimate goal is to prevent new infection, there is a need to evaluate condom use among FSW and the lessons will be vital for future HIV vaccine trial.

## Methods

Secondary data analysis of 2010 Integrated Bio-Behavioral Surveillance Survey was done with 4559 brothel and non-brothel FSWs in 9 Nigerian states. The survey involved HIV testing. Administered questionnaires had information on socio-demographic, HIV, sexual/reproductive health variables. Predictors of consistent condom use were evaluated using multiple logistic regression models.

## Results

The median age was 26years (15-49years); median age at first sex was 17years (11-22years); average clients/day was 4; 38% had ever been married with 8.1% currently married and 3.7% living with their spouse; 74.6% used condom consistently; 47.4% completed at least secondary education; 53.6% had been away from home for more than 1month; 60.2% tested and received HIV result within the last 12months; and 91.2% knew that condom could protect against HIV. Significant predictors of consistent condom use include testing and knowing result for HIV OR=1.9 95%CI:1.4-2.5; being away from home for ≥1month OR=1.4 95%CI:1.1-1.9; engaged in sex trade for 24months or less OR=2.7 95%CI:2.0-3.7; those that had 4 or more clients per day OR=1.7 95%CI:1.3-2.3) and those aged 25years and above OR=1.4 95%CI:1.1-2.1.

## Conclusion

Older FSW with shorter duration in sex trade, having more clients, aged above 25years and away from home are more likely to use condom consistently. Lessons from condom uptake among FSW may lead to targeting FSW with these features for inclusion criteria for HIV vaccine trial in Nigeria.

